# DEGnext: classification of differentially expressed genes from RNA-seq data using a convolutional neural network with transfer learning

**DOI:** 10.1186/s12859-021-04527-4

**Published:** 2022-01-06

**Authors:** Tulika Kakati, Dhruba K. Bhattacharyya, Jugal K. Kalita, Trina M. Norden-Krichmar

**Affiliations:** 1 Department of Epidemiology and Biostatistics, University of California, Irvine, Irvine, CA USA; 2 Department of Computer Science, Tezpur University, Assam, India; 3 Department of Computer Science, University of Colorado, Colorado Springs, Colorado Springs, CO USA

**Keywords:** Differentially expressed genes, Convolutional neural network, Classification, Transfer learning, Disease biomarkers

## Abstract

**Background:**

A limitation of traditional differential expression analysis on small datasets involves the possibility of false positives and false negatives due to sample variation. Considering the recent advances in deep learning (DL) based models, we wanted to expand the state-of-the-art in disease biomarker prediction from RNA-seq data using DL. However, application of DL to RNA-seq data is challenging due to absence of appropriate labels and smaller sample size as compared to number of genes. Deep learning coupled with transfer learning can improve prediction performance on novel data by incorporating patterns learned from other related data. With the emergence of new disease datasets, biomarker prediction would be facilitated by having a generalized model that can transfer the knowledge of trained feature maps to the new dataset. To the best of our knowledge, there is no Convolutional Neural Network (CNN)-based model coupled with transfer learning to predict the significant upregulating (UR) and downregulating (DR) genes from both trained and untrained datasets.

**Results:**

We implemented a CNN model, DEGnext, to predict UR and DR genes from gene expression data obtained from The Cancer Genome Atlas database. DEGnext uses biologically validated data along with logarithmic fold change values to classify differentially expressed genes (DEGs) as UR and DR genes. We applied transfer learning to our model to leverage the knowledge of trained feature maps to untrained cancer datasets. DEGnext’s results were competitive (ROC scores between 88 and 99$\%$) with those of five traditional machine learning methods: Decision Tree, K-Nearest Neighbors, Random Forest, Support Vector Machine, and XGBoost. DEGnext was robust and effective in terms of transferring learned feature maps to facilitate classification of unseen datasets. Additionally, we validated that the predicted DEGs from DEGnext were mapped to significant Gene Ontology terms and pathways related to cancer.

**Conclusions:**

DEGnext can classify DEGs into UR and DR genes from RNA-seq cancer datasets with high performance. This type of analysis, using biologically relevant fine-tuning data, may aid in the exploration of potential biomarkers and can be adapted for other disease datasets.

## Background

Transcriptomic profiling is important in understanding how genes regulate biological functions and control the underlying mechanisms of diseases. Differential expression (DE) analysis is used to identify the genes which undergo changes in biological patterns across healthy and disease conditions. This analysis can help researchers identify the differentially expressed genes (DEGs) which behave differently in disease conditions and help them prioritize these condition-specific genes as potential biomarkers for a particular disease. Numerous parametric and non-parametric statistical methods have been developed for DEG analysis based on logarithmic values of fold change (logFC) of gene expression in control state to disease state [1]. For instance, DESeq [2], DESeq2 [3], edgeR [4], and voom [5] use variance (dispersion) in gene expression values to identify the DEGs. However, due to the biases incorporated during computation of dispersion results, high false positive and false negative rates occur in predicting DEGs from RNA-seq data. Recently, many machine learning (ML) methods have been developed to classify genes on the basis of gene expression. For example, Support Vector Machine (SVM) with mutual information was used to classify genes that distinguish colon cancer patients from healthy patients [6]. Similarly, Logistic Regression (LR) has been used to classify gene expression from microarray experiments between acute lymphoblastic leukemia (ALL) and acute myeloid leukemia (AML) of Golub leukemia data, and between cervical cancer and normal tissues [7]. A Random Forest based method was proposed to classify genes in microarray data [8]. Additionally, an empirical study was carried out to assess various state-of-the-art supervised ML methods, namely Decision Tree (DTC), Linear Regression(LR), Naïve Bayes (NB), Random Forest (RFC), Support Vector Machine (SVC) in classifying gene expression in RNA-seq datasets [9]. However, these ML methods required selection of gene features as prior knowledge to train the classifier.

Deep learning (DL) coupled with transfer learning, on the other hand, has the ability to classify novel data by directly learning complex non-linear relationships among the features of the training data in one end-to-end classification system [10]. A Convolution Neural Network (CNN) is a type of deep learning, which applies mathematical convolutional approaches in one of more internal layers of the network. CNNs have architectures which enable massive computations and learning of non-linear relations between input and output data [11]. It has been recently demonstrated that the CNN is a powerful tool for classification in both image and non-image data because of characteristics, such as feature extraction, efficient hierarchical filtering with internal layers to deeply train a model, weight sharing capability to mitigate memory requirements, and utilization of neighborhood information [12–17]. For example, DeepInsight [17] is a CNN-based model, which has also shown promising results in feature extraction from non-image data, such as gene expression, text data, or synthetic data. Application of DL to RNA-seq data is challenging due to absence of appropriate labels and smaller sample sizes (*n*) as compared to the number of genes (*g*) [18]. Kakati et al. [19], proposed the first DL-based method to predict upregulating (UR) and downregulating (DR) genes from RNA-seq breast cancer and Parkinson’s disease datasets. Additionally, recent papers [20–22] have reported the use of transfer learning to predict cancer types or survival of cancer patients. Moreover, recently, many modified versions of ML based methods, such as SVC, RFC, and DTC have been reported which use learned knowledge to implement transfer learning between different domains of images [23–27]. However, with the increase in gene-expression data availability, there is the opportunity to create a generalized model which can use the trained features to identify potential biomarkers from UR and DR genes from small or large untrained datasets. To the best of our knowledge, currently there is no Convolutional Neural Network (CNN)-based model coupled with transfer learning to predict the gene expression directionality from both trained and untrained RNA-seq datasets.

In this paper, we propose a robust CNN-based model, DEGnext, in conjunction with transfer learning to classify the UR and DR genes from RNA-seq cancer datasets.

## Results

**Fig. 1:**
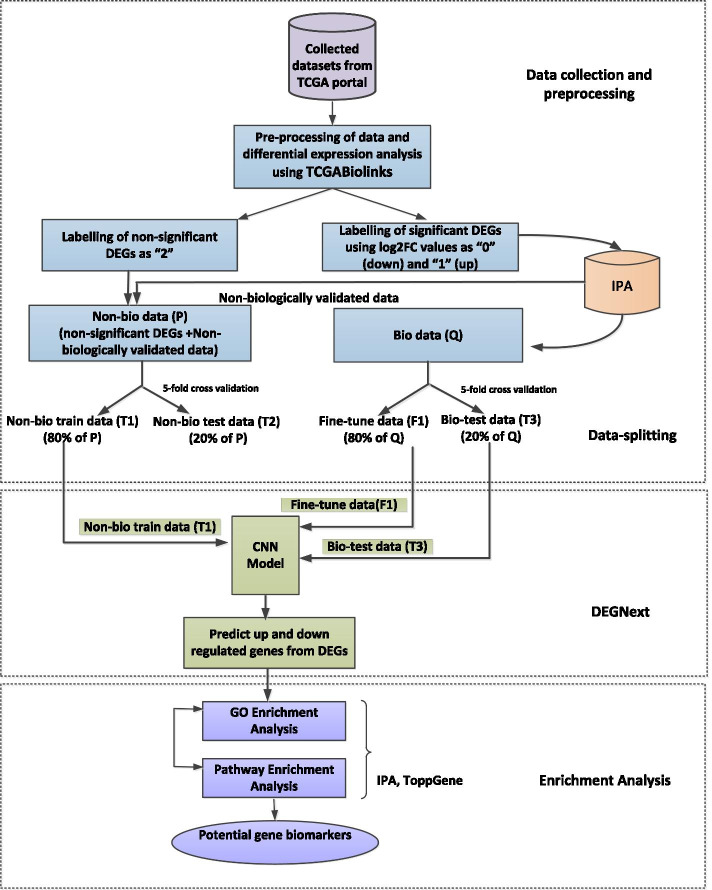
Workflow of DEGnext methodology. In this workflow, there are three main phases. The first phase involves data collection, preprocessing, labelling, and splitting of the data. Here, we split the data into two parts: non-biologically validated data (“non-bio data” or P) and the biologically validated data (“bio data” or Q). T1 is the non-biologically validated train data of P (“non-bio train data”, 80% of P). T2 is the non-biologically validated test data of P (“non-bio test data”, 20% of P). F1 is the fine-tune data of biologically validated data (“fine-tune data”, 80% of Q). T3 is the biologically validated test data of Q (“bio-test data”, 20% of Q). The second phase includes training (first level training) and fine-tuning (second level training) and testing of CNN model to predict UR and DR genes. The third phase includes downstream enrichment analyses of the predicted UR and DR genes to identify potential biomarkers related to a cancer dataset. The CNN architecture is illustrated in Fig. 2

**Fig. 2:**
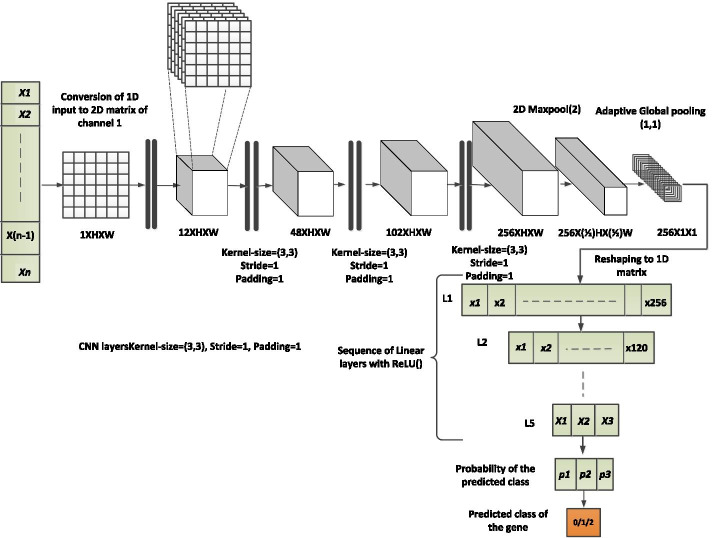
CNN architecture of DEGnext. The input to the model is a 1D input vector ($x_{1}$, $x_{2}$, $\dots$, $x_{n}$), which represents each gene row of a cancer dataset. This 1D vector is converted to a 2D matrix of channel 1 using np.reshape(). We used a sequence of eight 2D convolutional neural network (CNN) layers ($C_{1}$, $C_{2}$, $\dots$, $C_{8}$) with ReLU() as activation function. Each CNN layer uses kernel-size (3, 3), stride of 1, and padding equal to 1. We used a 2D Maxpool layer of kernel-size 2. In order to make the model inclusive for any input size, we used a 2D AdaptiveMaxPool layer with target output size of 1 $\times$ 1. The output of the CNN layers is fed to a sequence of 5 linear layers ($L_{1}$, $L_{2}$, $\dots$, $L_{5}$) with ReLU() as activation function. We used Softmax() to the output of linear layers, to find the probabilities of each class in the range of [0, 1]

In this section, we report the experimental results of DEGnext for both general and transfer learning. In the Methods section , we describe each step of the DEGnext workflow and CNN architecture in detail, so we will only give a brief overview here to aid in the interpretation of the results. Fig. 1 contains the workflow of DEGnext, while in Fig. 2, we illustrate the CNN architecture that we used in DEGnext to train and classify test data as UR and DR genes.

**Table 1:** Dataset abbreviations for cancer datasets used in DEGnext

Dataset abbreviation	Cancer type	Dataset abbreviation	Cancer type
BLCA	Bladder urothelial carcinoma	LIHC	Liver hepatocellular carcinoma
BRCA	Breast invasive carcinoma	LUAD	Lung adenocarcinoma
CHOL	Cholangiocarcinoma	LUSC	Lung squamous cell carcinoma
COAD	Colon adenocarcinoma	PRAD	Prostate adenocarcinoma
ESCA	Esophageal carcinoma	READ	Rectum adenocarcinoma
HNSC	Head and neck squamous cell carcinoma	STAD	Stomach adenocarcinoma
KICH	Kidney Chromophobe	THCA	Thyroid carcinoma
KIRC	Kidney renal clear cell carcinoma	UCEC	Uterine Corpus endometrial carcinoma
KIRP	Kidney renal papillary cell carcinoma	–	–

We used TCGABiolinks R package [28] to download 17 datasets (listed in Table 1).

For labeling the genes in the datasets, we used logFC values in addition to disease-related knowledge from Ingenuity Pathway Analysis (IPA) to divide the data into biologically or non-biologically validated data. First, we use general learning on DEGnext, to predict the directionality of DEGs from all 17 datasets. Second, we divide 17 datasets into 9 training datasets and 8 testing or untrained datasets and use transfer learning to leverage the knowledge (features, weights) acquired from the previously trained DEGnext model to predict UR and DR genes from rest 8 testing datasets. Third, we evaluate the performance of the DEGnext model for general and transfer learning against five ML methods, namely Decision Tree (DTC), Linear Regression(LR), Naïve Bayes (NB), Random Forest (RFC), Support Vector Machine (SVC), and XGBoost in terms of accuracy, recall, precision, F-measure, Matthews correlation coefficient (MCC), and Receiver Operating Characteristic (ROC) scores. Fourth, we test the robustness of DEGnext by augmenting the datasets with seven levels of Gaussian noise data (1, 10, 50, 100, 500, 1000, 1500) and compare it with other ML methods. Fifth, we obtain the Gene Ontology (GO) term enrichment and pathway enrichment of the predicted up/down regulated genes from cancer datasets. Finally, we identify the potential biomarkers mapped to the significant pathways related to BRCA and UCEC datasets. Throughout the text and figures, we will be using the following abbreviations for the testing and training data. The non-biologically validated data is labeled as P (“non-bio data”), and the biologically validated is labeled as Q (“bio data”). The P data were split as non-biologically validated train data (“non-bio train data” or T1) and non-biologically validated test data (“non-bio test data” or T2) in the ratio of 80:20. Similarly, the Q data were split in the ratio of 80:20 as fine-tune (F1) and biologically-validated test data (“bio-test data” or T3), respectively.

### Performance of DEGnext in general learning experiment

In the general learning experiment, we first trained the model with non-bio train data (T1) for all 17 datasets with fivefold cross-validation. Then, we took the best fold models for each dataset and further trained the model with fine-tune data (F1). To evaluate the overall DEGnext output quality in general learning, we calculated the mean of five metrics, namely accuracy, recall, precision, F-measure, and MCC across fivefolds of bio-test data (T3). In Table 2, we report the performance of DEGnext on bio-test data (T3) for all 17 datasets. We find that the mean of all the three metrics, namely accuracy, recall, and precision scores for all the datasets was within the range of 95-100$\%$. The F-measure and MCC scores for all the 17 datasets were above 0.85. This demonstrates that the proposed model is effective in classifying the up/down regulated genes from bio-test data of the respective TCGA cancer datasets. For instance, the MCC score for datasets CHOL, KICH, KIRC, KIRP, LUAD, LUSC, and THCA is 1, which signifies perfect prediction of up/down regulated genes by DEGnext.

**Table 2:** Performance of DEGnext on bio-test data (T3) of all 17 datasets using general learning considering fivefold cross validation

Dataset	Accuracy	Recall	Precision	F-measure	MCC
BLCA	98.42	98.42	98.49	0.98	0.97
BRCA	98.80	98.80	98.83	0.99	0.98
	100.00	100.00	100.00	1.00	1.00
CHOL COAD	99.64	99.64	99.65	1.00	0.99
ESCA	97.95	97.95	98.10	0.98	0.96
HNSC	99.32	99.32	99.34	0.99	0.98
KICH	100.00	100.00	100.00	1.00	1.00
KIRC	99.78	99.78	99.78	1.00	1.00
KIRP	100.00	100.00	100.00	1.00	1.00
LIHC	95.93	95.93	96.23	0.96	0.85
LUAD	99.82	99.82	99.83	1.00	1.00
LUSC	99.88	99.88	99.88	1.00	1.00
PRAD	99.35	99.35	99.36	0.99	0.99
READ	95.39	95.39	96.54	0.95	0.92
STAD	96.89	96.89	97.06	0.97	0.93
THCA	99.87	99.87	99.87	1.00	1.00
UCEC	99.60	99.60	99.61	1.00	0.99

### Performance of DEGnext in transfer learning

General learning results for DEGnext are nearly perfect. However, most RNA-seq datasets do not have appropriate labels and have smaller sample sizes (n) compared to number of genes (g). In those situations, a general model cannot be obtained using supervised learning and we must rely on models trained on another datasets. This motivated us to make DEGnext generalizable for RNA-seq datasets, irrespective of dataset size or appropriate labels. Instead of training a CNN from scratch for any new dataset, we wanted to use the pretrained DEGnext model on new datasets without labels to predict significant UR and DR genes. Transfer learning can leverage the knowledge of trained feature maps from trained model to untrained cancer datasets . Moreover, general learning on 17 datasets is time-consuming since we need to train and fine-tune the model for each dataset separately to predict UR and DR genes from bio-test data of respective dataset. In order to analyze the effectiveness of DEGnext in transfer learning, we divided the 17 datasets into two groups based on the sample sizes: 9 training datasets with large size (BRCA, LIHC, LUAD, LUSC, KIRC, KIRP, PRAD, THCA, and UCEC) and 8 testing or untrained datasets with smaller size (BLCA, CHOL, COAD, ESCA, HNSC, KICH, READ, and STAD). We used this dataset splitting strategy so that features learned during training generalized to any unknown or new dataset regardless of size. The testing datasets comprised of 100$\%$ of biologically validated data (Q). Since the testing datasets are smaller in size than the training datasets, we first use non-bio train data (T1) and fine-tune data (F1) to train DEGnext model on all 9 training datasets sequentially. The trained model is then tested on biologically validated data (Q) of the untrained datasets to predict UR and DR genes from untrained datasets.

From Table 3, we show that except for COAD and READ, all other TCGA untrained datasets, attain an overall performance above 84$\%$ when using our DEGnext model on biologically validated data (Q).

**Table 3:** Performance of DEGnext on biologically validated data (Q) of 8 testing or untrained datasets using transfer learning

Dataset	Accuracy	Recall	Precision	F-measure	MCC
BLCA	95.69	95.69	95.76	0.96	0.91
CHOL	98.26	98.26	98.49	0.98	0.94
COAD	84.21	84.21	88.44	0.84	0.72
ESCA	92.97	92.97	94.68	0.93	0.61
HNSC	98.44	98.44	98.49	0.98	0.96
KICH	98.75	98.75	98.79	0.99	0.97
READ	86.05	86.05	89.23	0.86	0.75
STAD	97.77	97.77	97.89	0.98	0.95

The MCC scores for these datasets, such as BLCA, CHOL, HNSC, KICH, and STAD was above 0.9, which signified the better prediction of UR and DR genes from the untrained datasets. This process leverages the optimization and reduces the amount of data and time required to train new models for new datasets. Thus, we conclude that DEGnext was able to transfer the knowledge of learned feature maps from the trained datasets to untrained datasets effectively.

### Comparison of DEGnext performance with other ML methods

**Fig. 3:**
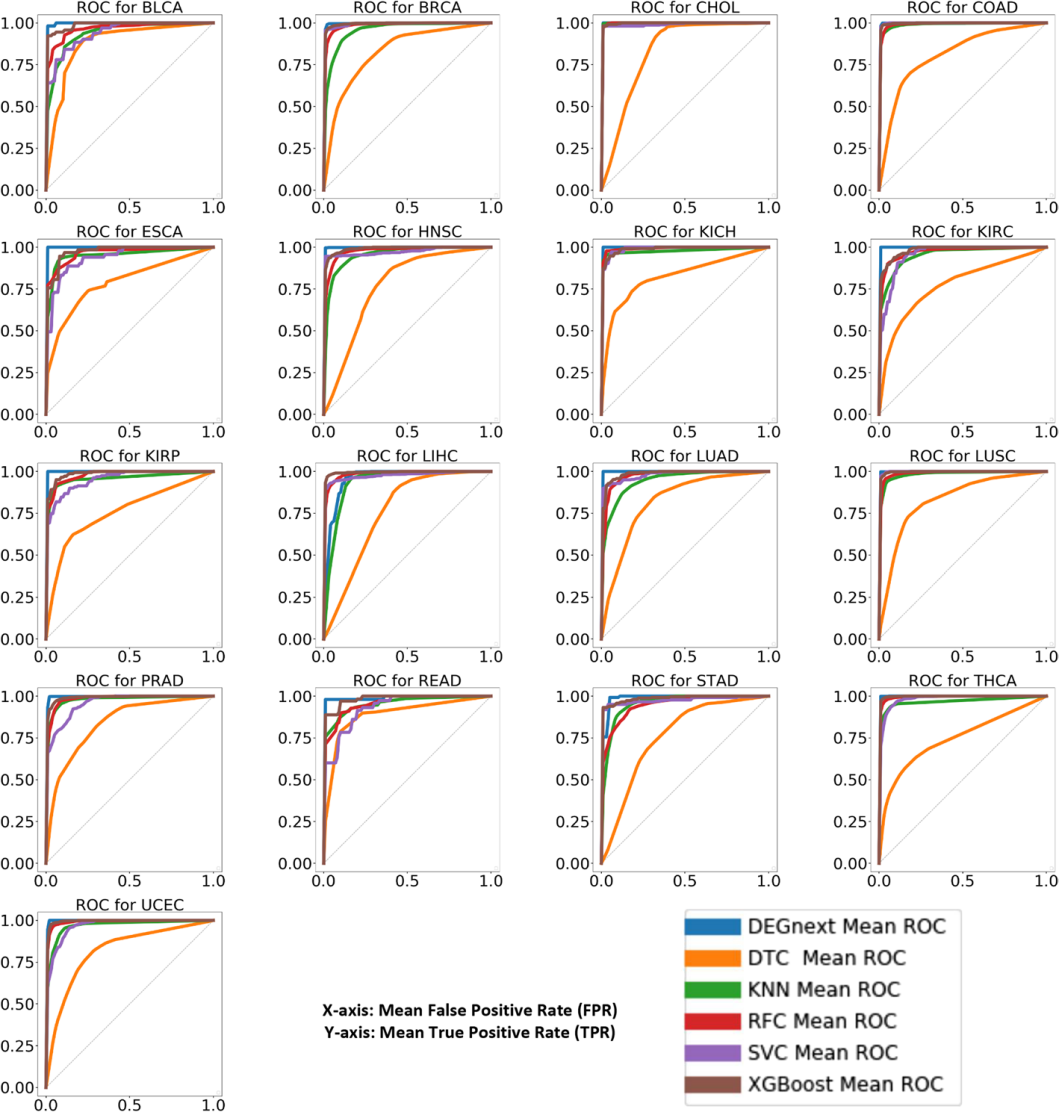
ROC curves of 17 datasets for general learning. Comparison of ROC scores for general learning of bio-test data (T3) for all 17 datasets

We assessed the performance of DEGnext for both general and transfer learning in predicting UR and DR against five ML methods. In particular, we compared DEGnext to DTC, KNC, RFC, SVC, and XGBoost in terms of mean of accuracy, recall, precision, F-measure, MCC, and ROC scores. For general learning, we trained and fine tuned DEGnext and the ML methods with non-bio train data (T1) and fine-tune data (F1) for each dataset. Then, we tested the models with bio-test data (T3) of all 17 datasets with fivefold cross validation. In Additional file 1: Table S1, we see that DEGnext’s results on general learning are competitive with the other ML methods for all 17 datasets. In Fig. 3, ROC scores for all 17 datasets were 0.96 or above. Similarly, we found XGBoost outperformed for most of the datasets as compared to other traditional ML-methods, such as DTC, KNC, and SVC.

**Fig. 4:**
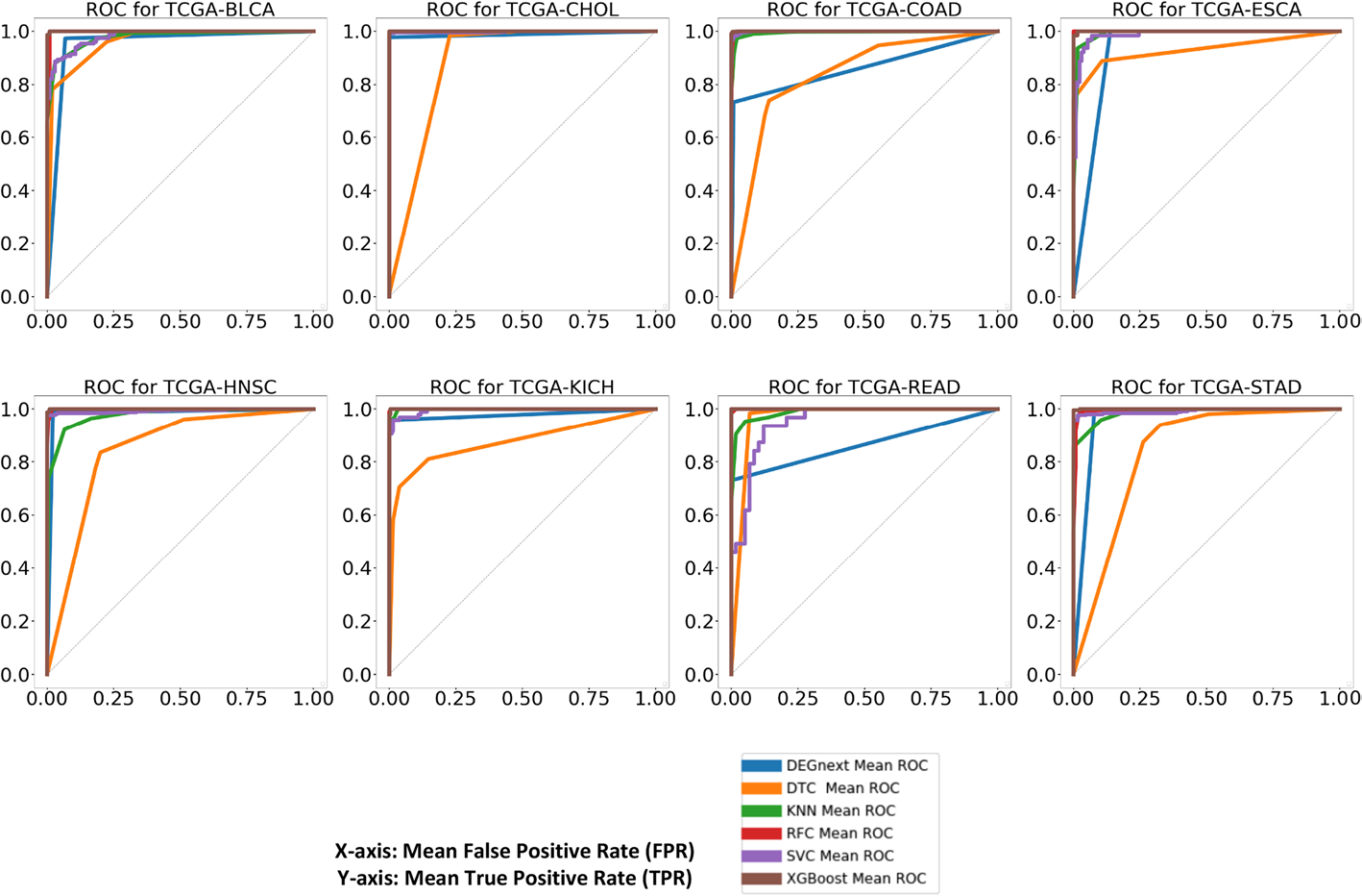
ROC curves of 8 untrained datasets for transfer learning. Comparison of ROC scores for transfer learning on 100$\%$ of bio data data (Q) for all test datasets, namely BLCA, CHOL, COAD, ESCA, HNSC, KICH, READ, and STAD

For transfer learning, we trained DEGnext and the ML methods on 80$\%$ of non-bio train data (T1) and 80$\%$ of fine-tune data (F1) of the 9 training datasets sequentially. We then tested the pretrained model on 100$\%$ of the biologically validated data (Q) of the untrained datasets. In Additional file 1: Table S2, we show that except for COAD and READ datasets, DEGnext’s results are consistent with those of the other ML methods in terms of accuracy, recall, precision, F-measure, MCC, and ROC-scores for all the untrained datasets. In Fig. 4, the ROC scores were above 0.85 for DEGnext and XGBoost for all testing datasets in line with to other ML methods. Therefore, from our results, we can conclude that DEGnext is competitive or better in both general learning and transfer learning.

### Robustness of DEGnext

**Fig. 5:**
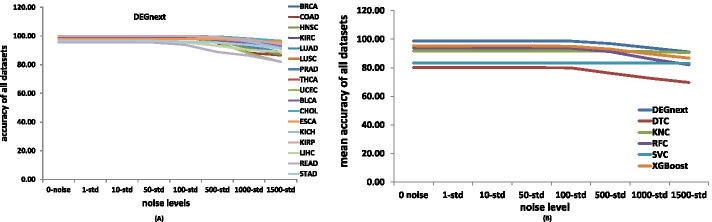
Robustness comparison for DEGnext. **A** Robustness of DEGnext to noisy data for bio-test data (T3) of all the 17 datasets. **B** Comparison with other ML methods in terms of the mean accuracy for bio-test data (T3) of all the 17 datasets in presence of different levels of Gaussian noise

To demonstrate that DEGnext is robust to noise, we tested the performance of DEGnext with increasing Gaussian noise in 7 levels: 1$\%$, 10$\%$, 50$\%$, 100$\%$, 500$\%$, 1000$\%$, and 1500$\%$. In Fig. 5A, we show that for all the datasets, DEGnext was quite robust to the increasing levels of noise up to 500$\%$ standard deviation. However, for READ, COAD, and UCEC datasets, the accuracy decreased with the increase levels of noise. On the other hand, in Fig. 5B, we show that DEGnext performs competitively better in terms of the mean accuracy for bio-test data (T3) of all the 17 datasets.

### GO enrichment analysis of predicted UR and DR genes

After classifying the DEGs into UR and DR genes using our DEGnext model, we assessed the GO enrichment of the predicted UR and DR genes using ToppGene Suite. As shown in Table 4, the predicted UR and DR genes were enriched with some common GO terms associated with carcinogenesis.

**Table 4:** Analysis of GO enrichment of predicted UR and DR genes for BRCA and UCEC datasets

Dataset	GO ID/attribute	***p*** value	***q*** value
BRCA	Cellular component morphogenesis	1.72E−06	8.39E−03
	Cellular response to endogenous stimulus	5.19E−06	8.39E−03
	Cell adhesion	5.32E−06	8.39E−03
	Biological adhesion	6.15E−06	8.39E−03
	Cell morphogenesis	9.77E−06	1.07E−02
	Negative regulation of response to stimulus	1.25E−05	1.14E−02
	Negative regulation of intracellular signal transduction	4.77E−05	3.72E−02
UCEC	Reproductive process	1.19E−05	2.61E−02
	Reproduction	1.24E−05	2.61E−02
	Positive regulation of plasminogen activation	3.18E−05	4.46E−02

For instance, we observed that the predicted UR and DR genes from BRCA datasets were related to GO terms such as *cellular adhesion* and *cell morphogenesis*, which are associated with cancer cell invasion and metastasis [29]. On the other hand, for the UCEC dataset, the GO terms mapped from the predicted UR and DR genes were mainly focused on *reproductive processes*, *reproduction*, and *positive regulation of plasminogen activation* and had more significant *p*-values and *q*-values. There is evidence [30] that activation of plasminogen from cancer cells leads to breakdown of cellular components, which in turn leads to invasion of cancer cells into other areas of the body. These results suggest that the predicted UR and DR genes for the breast and uterine cancer datasets were functionally enriched with significant GO terms with lower *p*-values and *q*-values associated with cancer.

### Pathway enrichment analysis of predicted UR and DR genes

**Table 5:** Ten significant pathways mapped from predicted UR and DR genes of BRCA and UCEC

Cancer	Ingenuity canonical pathways	Mapped predicted UR genes	Mapped predicted DR genes
BRCA	RhoGDI signaling	ARHGEF17, CDH18, CDH5, FNBP1, PPP1R12C, RDX, RHOQ	CREBBP, RHOB, CD44, CDH6, SRC, ESR1, RAC1
	ILK signaling	CCND1, FNBP1, ITGB7, MYH6, RHOQ, VIM	CREBBP, MYH11, CREB3, RHOB, IRS2, ACTN2, RAC1
	Glioblastoma multiforme signaling	CCND1, FNBP1, FZD7, ITPR1, PLCZ1, RHOQ	CDK6, CDKN1A, PLCH2, RHOB, SRC, RAC1
	Leukocyte extravasation signaling	CDH5, PRKCG, RDX, VCAM1	PRKCH, CLDN12, CD44, SRC, MMP27, CLDN2, RAP1GAP, ACTN2, RAC1
	Wnt/$\beta$-Catenin signaling	CCND1, CDH5,FZD7, PIN1, TGFBR1, TLE4	CREBBP, CD44, SRC, CSNK1D, DVL3, POU5F1
	Cholecystokinin/gastrin-mediated signaling	FNBP1, ITPR1, PRKCG, RHOQ	PRKCH, RHOB, SRC, CCKBR, RAC1
	Factors promoting cardiogenesis in vertebrates	CCND1, FZD7, MYH6, PLCZ1, PRKCG, TGFBR1	CREBBP, CREB3, PRKCH, PLCH2
	Wnt/Ca+ pathway	FZD7,PLCZ1	CREBBP, CREB3, PLCH2, DVL3
	Dopamine-DARPP32 feedback in cAMP signaling	GRIN2D, ITPR1, PLCZ1, PRKCG	CREBBP, CREB3, PRKCH, PLCH2, CSNK1D, CACNA1S
	UVC-induced MAPK signaling	PRKCG, SMPD1	PRKCH, ARAF, SRC
UCEC	PTEN signaling	ITGA4, MCRS1, SOS1	INPP5K, CBL
	Ephrin receptor signaling	ATF4, ITGA4, SOS1	CREBBP, EPHA6
	Integrin signaling	ACTN1, CAPN2, ITGA4, SOS1	ZYX, ITGA2B, ITGB8
	ERK/MAPK signaling	ATF4, DUSP9, ITGA4, KSR1, NFATC1,SOS1	CREBBP
	PPAR signaling	PPARA, SOS1	CREBBP, TNFRSF11B, NCOR2
	FLT3 signaling in hematopoietic progenitor cells	ATF4, SOS1	CREBBP, CBL
	Calcium signaling	ATF4, ATP2B1, MYH10, NFATC1	CREBBP, CACNA1C
	ILK signaling	ACTN1, ATF4, MYH10	CREBBP, ITGB8
	B Cell receptor signaling	ATF4, NFATC1, SOS1	CREBBP, INPP5K
	IL-6 signaling	ABCB1, SOS1	TNFRSF11B, CYP19A1

We performed a pathway enrichment analysis of the predicted UR and DR genes obtained from the bio-test data of BRCA and UCEC datasets using IPA. In Table 5, we report 10 significant pathways, mapped from predicted UR and DR genes of BRCA and UCEC, associated with progression of breast and cancer datasets. We discuss these below.

1. BRCA dataset
  - RhoGDI signaling: The main functions of the Rho family of GTPase involve promoting cellular adhesion, proliferation, and metastasis of breast cancer cells. RhoB exerts positive effects on increasing expression of estrogen receptor alpha (ER$\alpha$) and progesterone receptor (PR), which correlate to the progression of breast cancer [31]. Pathway analysis shows that predicted upregulating genes such as RHOQ, CDH5, and FNBP1 are associated with signaling by Rho family GTPase. There is evidence in prior research that Cadherin-5 (CDH5) is a potential biomarker for metastasis of breast cancer [32]. The modulation of RHOB and RHOG regulates the proliferation and differentiation of cancer cells, which influences the prognosis of breast cancer [33].
  - ILK pathway: Activation of oncogenes leads to overgrowth of cancer cells, which is the hallmark of the progression of a malignant tumor, like breast cancer. Over-expression of integrin-linked-kinase (ILK) promotes proliferation and growth of breast cancer cells [34]. Under normal conditions, ILK is involved in adhesion of cells, homeostasis of tissue and other critical cardiac functions. It has been found that upregulation of ILK leads to significant acceleration of tumor development in breast cancer. From the bio-test gene list, we find that ITGB7, which is predicted to be up-regulated by DEGnext is responsible for altered ILK pathway. It leads to abnormal cell proliferation in breast cancer [35]. Similarly, DEGnext predicts upregulation of RHOB, which has been reported to exert positive effects during carcinogenesis of breast tumors [33].
2. UCEC dataset
  - PTEN signaling: We found that within the significant PTEN signaling pathway that some mapped genes, such as ITGA4, MCRS1, and SOS1 were predicted to be upregulated by the DEGnext model. In [36], it has been reported that ITGA4 is a potential target for carcinogenesis because overexpression of ITGA4 promotes invasion of tumor cells and metastasis. Similarly, in [37], it was found that genes like MCRS1 are overexpressed in the advanced stage of cervical cancer. Additionally, in [38], the authors confirmed that overexpression of the SOS1 gene correlates with the progression of cancer.
  - Ephrin receptor signaling: In a significant pathway called the Ephrin Receptor Signaling pathway, we found several mapped genes, namely ATF4, EPHA6, ITGA4, and SOS1, which were predicted to be upregulated by DEGnext and were related to carcinogenesis. For example, the gene Erythropoietin-producing human hepatocellular (EPH) receptors, such as EPHA6 has pro-tumorigenic effects and induces a number of cellular processes, such as adhesion, proliferation, differentiation during carcinogenesis of cervical cancer [39].

## Discussion

We developed a CNN model called DEGnext to classify UR and DR genes from the DEGs of TCGA RNA-seq cancer datasets. We established that transfer-learning combined with the DEGnext model made the model effective in classifying UR and DR genes from untrained datasets. We compared the performance of DEGnext with 5 other ML methods, and DEGnext is competitive in terms of accuracy, sensitivity, specificity, F-measure, MCC, and ROC scores. In particular, for general learning, in Fig. 3 and in Additional file 1: Table S1, we show that DEGnext performs competitively or better than other existing ML methods for all 17 datasets. For transfer learning, in Fig. 4 and in Additional file 1: Table S2, we show that except for the COAD and READ datasets, DEGnext prediction results are better than existing ML methods. For the COAD and READ datasets, a similar discrepancy in precision and recall has been reported in the results for models based on CNNs [12, 13]. The DEGnext model was robust in terms of accuracy and was able to withstand the addition of Gaussian noise.

We validated the biological enrichment of the predicted UR and DR genes from the BRCA and UCEC datasets in terms of GO and pathway enrichment. We found that the predicted UR and DR genes were enriched with GO terms related to cancer with significant *p*-values and *q*-values. Similarly, for biological pathways, we found that the predicted UR and DR genes were enriched in pathways associated with breast cancer, such as the ILK pathway and the Rho GTPase signaling pathway. Pathways mapped from the predicted UR and DR genes of the UCEC dataset also play significant roles in carcinogenesis of cervical cancer such as PTEN signaling and Ephrin receptor signaling pathways.

## Conclusions

The proposed CNN model, DEGnext provides a novel approach for prediction of UR and DR genes from both trained and untrained datasets using both logFC values and disease-related biological knowledge. The downstream analysis of the predicted UR and DR genes has provided insights into the underlying mechanisms and aided in the identification of the prime regulators of carcinogenesis of breast cancer and uterine cancer. Therefore, through the prediction and classification of DEGs, DEGnext may aid in the exploration of potential biomarkers of a disease from other RNA-seq datasets.

## Methods

### Dataset collection and preprocessing

We used TCGABiolinks R package [28] to download 17 datasets (listed in Table 1) from TCGA portal. Figure 1 is a schematic depiction of the workflow of the methodology which is described below.

1. Although each of the 17 downloaded datasets had different sample sizes, there were 60,483 mRNA transcripts in each dataset. We used the following queries and data categories: Transcriptome Profiling; data type: Gene Expression Quantification; workflow type: HTSeq-Counts; sample type: Primary Tumor and Solid Tissue Normal; legacy: FALSE, to download the cancer datasets from the TCGA portal. First, we prepared the dataset to represent it as an expression matrix with genes as rows and samples as columns. Out of 60,483 mRNA transcripts, we obtained 56,493 mRNA transcripts, which were mapped to the human genome (Genome Reference Consortium Human Build 38, GRCh38).
2. We preprocessed the gene expression data using *TCGAanalyze_Preprocessing()* with a gene expression cut off threshold = 0.6 and found that 56,493 number of mRNA transcripts above this threshold were within the inter-quartile range.
3. We mapped the ENSEMBL identifiers of the mRNA transcripts, and kept only those mRNA transcripts which had valid HGNC gene symbols. From the 56,493 mRNA transcripts, we found that 37,614 genes had valid HGNC symbols.
4. In RNA-seq data, different sample conditions have different sequencing depths and RNA compositions, which may cause complications in downstream analysis [40]. We performed data normalization using *TCGAanalyze_Normalization()* to adjust several gene-level effects, such as GC-content and sequencing depth. Internally, *TCGAanalyze_Normalization()* utilizes the EDASeq package [41] to perform within-lane normalization and between-lane normalization [42]. 8686 genes remained after normalization for these gene-level effects.
5. For each of the 17 datasets, we filtered the genes using *TCGAanalyze_Filtering()*, with a quantile cut off 0.25 and found 6514 filtered genes (FG) above this threshold. We used a strict parameter cut off for preprocessing and filtering of the genes before DE analysis, because the main objective of our approach is to find significant predicted UR and DR genes related to cancer progression.
6. We used *TCGAanalyze_DEA()* to perform DE analysis on the filtered genes (FG) across normal versus tumor conditions with a false discovery rate (FDR) cutoff 0.01, yielding significant labeled DEGs (SDEGs) for each cancer dataset. Out of the 6514 FGs, the non-significantly differentially expressed for FDR cutoff of 0.01 were labeled “2” as neutral genes.
7. The number of SDEGs for each dataset was different as shown in Table 6. Next, we labeled the SDEGs on the basis of logFC threshold = 0. That is, if the logFC value of a SDEG was below 0, then the DEG was labeled as “0” for down regulated (DR) gene. If it was above 0, then the DEG was labelled as “1” for up-regulated (UR) gene.
8. We input the SDEGs for each dataset into the IPA tool [43] to check if they were related to the specific cancer. We found that, between 4 and 47$\%$ of the significant DEGs were related to the respective cancer disease and we categorized them as biologically validated data (“bio data” or Q). The neutral genes and non-biologically validated genes together formed the remaining data (“non-bio data” or P).
9. We split P and Q data as shown in Fig. 1. The non-bio data (P) were split as non-bio train data (T1) and non-bio test data (T2) in the ratio of 80:20. Similarly, the bio data (Q) were split in the ratio of 80:20 as bio-test data (T3) and fine-tune (F1) data. In order to avoid bias, we considered fivefold cross validation to test the model.
10. We performed two experiments: one for general training and testing and the other experiment for transfer learning. We leveraged the knowledge (features, weights) learned from the previously trained DEGnext model to predict UR and DR genes from 8 untrained datasets.
11. For general learning, we tested the effectiveness of DEGnext model to classify UR and DR genes from respective bio-test data (T3) of all 17 datasets.
12. We checked the generalizability of DEGnext by using transfer learning of the significant feature-maps into bio data (Q) of 8 untrained datasets.
13. We compared the performance of our DEGnext against five ML methods, DTC, KNC, RFC, SVC, and XGBoost in terms of accuracy, recall, precision, F-measure, MCC, and ROC scores.
14. We tested the robustness of DEGnext by augmenting the datasets with seven levels of Gaussian noise data (1, 10, 50, 100, 500, 1000, 1500) and compared results with other ML methods.
15. We used two tools, ToppGene Suite and IPA, for Gene Ontology (GO) and pathway enrichment analysis of the predicted UR and DR genes for BRCA and UCEC datasets, respectively.

In Table 6, for each dataset, we show the size of preprocessed and filtered datasets, number of significant labeled DEGs (sDEGs), number of genes in non-bio train data (T1), non-bio test data (T2), fine-tune data (F1), and bio test data (T3).

**Table 6:** Number of genes and samples from preprocessed and filtered gene expression data used in labeling, training, fine-tuning, and testing

Dataset	Filtered genes (FG)$\#$gene$\times$ $\#$normal samples $\#$ tumor samples	Significant labeled DEGs (SDEGs)	Bio genes(Q)	Non-bio train data(T1)	Non-bio test(T2)	Fine-tune(F1)	Bio-test(T3)
BRCA	6514$\times$ 113 1102	4939	2327	3349	838	1861	466
BLCA	6514$\times$ 19 414	2496	254	5008	1252	203	51
CHOL	6514$\times$ 9 36	2811	552	4768	1193	441	111
COAD	6514$\times$ 41 478	4213	1399	4092	1023	1119	280
ESCA	6514$\times$ 11 161	1420	193	5056	1265	154	39
HNSC	6514$\times$ 44 500	3860	734	4624	1156	587	147
KICH	6514$\times$ 24 65	3422	306	4966	1242	244	62
KIRC	6514$\times$ 72 538	4822	455	4847	1212	364	91
KIRP	6514$\times$ 32 288	3535	337	4941	1236	269	68
LIHC	6514$\times$ 32 288	4372	1498	4012	1004	1198	300
LUAD	6514$\times$ 59 533	4387	566	4758	1190	452	114
LUSC	6514$\times$ 49 502	4833	839	4540	1135	671	168
PRAD	6514$\times$ 52 498	3803	1080	4347	1087	864	216
READ	6514$\times$ 10 166	2678	121	5114	1279	96	25
STAD	6514$\times$ 32 375	3379	388	4900	1226	310	78
THCA	6514$\times$ 58 502	4292	3031	2786	697	2424	607
UCEC	6514$\times$ 35 551	3992	999	4412	1103	799	200

Q: bio data; T1: non-bio train data; T2: non-bio test data; F1: fine tune data; T3: bio test data

### DEGnext model construction and implementation

The DEGnext is a CNN model proposed to predict UR and DR genes from RNA-seq cancer datasets. We implemented the CNN model using Pytorch in Python DL platform [44], as shown in Fig. 2.

The input to the model is a 1D input vector ($x_{1}$, $x_{2}$, $\dots$, $x_{n}$), which represents each gene row of the cancer dataset. This 1D vector is converted to a 2D matrix of channel 1 using np.reshape() function. We used a sequence of eight 2D convolutional neural network (CNN) layers ($C_{1}$, $C_{2}$, $\dots$, $C_{8}$) with ReLU() as activation function. Each CNN layer uses kernel-size (3, 3), stride of 1, and padding equal to 1. We used a 2D Maxpool layer of kernel-size 2. In order to make the model inclusive for any input size, we used a 2D AdaptiveMaxPool layer with target output size of 1 $\times$ 1. The output of the CNN layers is fed to a sequence of 5 linear layers ($L_{1}$, $L_{2}$, $\dots$, $L_{5}$) with ReLU() as activation function. In DEGnext, we have used the activation output from the last linear layer as feature representation and applied Softmax() to find the probabilities of each class in the range of [0, 1]. The values for the key hyperparameters are listed in Table 7.

**Table 7:** Values of hyperparameters used in DEGnext model

Hyperparameters	First level training	Fine-tuning
Epoch	50	31
Loss function	CrossEntropyLoss()	BCEWithLogitsLoss()
Learning-rate	1e−4	1e−4
Betas	(0.9, 0.999)	(0.9, 0.999)
eps	1e−08	1e−08
Weight-decay	0	0
Batch-size	256	64

We performed two experiments to test the effectiveness of our model.

*Experiment 1 (General Learning):* In the first experiment, we used all 17 cancer datasets to train, fine-tune and test the corresponding bio-test data (T3) from each dataset. Since the non-bio train data (P) has three labels ‘0’, ‘1’, and ‘2’, this training is for a three-class problem. For the first-level of training, DEGnext runs for 50 epochs with a batch size of 256 and it uses *CrossEntropyLoss()* as a loss function and *optim.Adam()* as an optimizer to compute the cross entropy loss between the output ($y^{pred}$) for a given input *x* and updates the parameters based on the gradients. For predicted classes 0, 1, 2, the input gene is classified as DR, UR or neutral gene.

For the second level of training, we use fine-tune data (F1) on the best model from first level of training for each dataset. Since fine-tune data (F1) have ‘0’ and ‘1’ labels, the second level of training is a two-class problem. Here, we used the *BCEWithLogitsLoss()* loss function to fine-tune the model with a batch size of 64 for each dataset. After training for 31 epochs, the respective models are then tested using bio-test data (T3) of each dataset. The second level of training incorporates both prior disease-related biological knowledge and log2FC estimates (sample variance) of the data to the CNN model, which enables capture of non-linear gene expression patterns and enhances prediction performance of the model in determining UR and DR genes. The major advantage of our CNN model is that it allows performing very efficient transfer learning by reusing the feature-map signatures learned from the trained model.

*Experiment 2 (Transfer learning): *For the second experiment, we divided 17 datasets into two groups: training datasets (BRCA, LIHC, LUAD, LUSC, KIRC, KIRP, PRAD, THCA, and UCEC) and testing datasets (BLCA, CHOL, COAD , ESCA, HNSC, KICH, READ, and STAD). The training datasets are larger in size than the testing datasets. The testing datasets comprised of 100 $\%$ of biologically validated data. We choose the best fold data for each dataset and trained on 80$\%$ of non-bio train data (T1) of all 9 training datasets one after another with a batch size of 64. For training, since non-bio train data (T1) has three labels, ‘0’, ‘1’, and ‘2’, we used *CrossEntropyLoss()* as a loss function and *optim.Adam()* as an optimizer, with a batch size of 256 to train the model on the 9 training datasets one after another. For fine-tune, all we needed to do was to customize and modify the output layer L5 and remove the final softmax layer to classifying the DEGs as ‘0’ or ‘1’. We used the *BCEWithLogitsLoss()* loss function to fine-tune the model again with the fine-tune data (F1) for all 9 training datasets. For testing, we did not retrain the model, but instead used the pretrained model to predict UR and DR genes from all 8 testing datasets.

## Supplementary information

**Additional file 1.** Comparison of ROC scores of DEGnext with other ML methods for general learning and transfer learning.

**Additional file 2.** Readme.

**Additional file 3.** Source codes for general learning.

**Additional file 4.** Source codes for transfer learning.

**Additional file 5.** Source codes for test case.

## Data Availability

TCGA cancer datasets can be downloaded from TCGA portal using TCGABiolinks R package. All codes and the preprocessed datasets can be found in https://github.com/tulikakakati/DEGnext. The preprocessed datasets can be found in “datasets” folder of the shared link. Additional file 2: Readme.pdf file. The python scripts for general learning and transfer learning can be found as zip folders in Additional files: 3 and 4, respectively. Additional file 5: zip folder with trained and fine-tuned DEGnext model ready to test on an input file dataset.

## Abbreviations

ALL: Acute lymphoblastic leukemia
AML: Acute myeloid leukemia
BLCA: Bladder urothelial carcinoma
BRCA: Breast invasive carcinoma
CHOL: Cholangiocarcinoma
CNN: Convolutional neural network
COAD: Colon adenocarcinoma
DE: Differential expression
DEGs: Differentially expressed genes
DL: Deep learning
DR: Downregulating
DTC: Decision tree
ESCA: Esophageal carcinoma
FG: Filtered genes
F1: Fine-tune data
GO: Gene ontology
HNSC: Headand neck squamous cell carcinoma
IPA: Ingenuity pathway analysis
KICH: Kidney chromophobe
KIRC: Kidney renal clear cell carcinoma
KIRP: Kidney renal papillary cell carcinoma
KNC: K-nearest neighbors
LIHC: Liver hepatocellular carcinoma
logFC: Logarithmic fold change
LUAD: Lung adenocarcinoma
LUSC: Lung squamous cell carcinoma
MCC: Matthews correlation coefficient
ML: Machine learning
P: Non-biologically validated data (non-bio data)
PRAD: Prostate adenocarcinoma
Q: Biologically validated data (bio data)
READ: Rectum adenocarcinoma
RFC: Random forest
ROC: Receiver operating characteristic
SDEGs: Significant labeled DEGs
STAD: Stomach adenocarcinoma
SVC: Support vector machine
TCGA: The cancer genome atlas
THCA: Thyroid carcinoma
T1: Non-bio train data
T2: Non-bio test data
T3: Bio-test data
UCEC: Uterine corpus endometrial carcinoma
UR: Upregulating
