# Supplementary material for: DEGnext: classification of differentially expressed genes from RNA-seq data using a convolutional neural network with transfer learning
Source: BMC Bioinformatics. 2022 Jan 6;23:17. doi: 10.1186/s12859-021-04527-4 (PMC8734099; doi:10.1186/s12859-021-04527-4)
Supplement: Supplementary file 1 — Additional file 1. Comparison of ROC scores of DEGnext with other ML methods for general learning and transfer learning. [file 12859_2021_4527_MOESM1_ESM.pdf]

Table S1: ROC scores of DEGnext and ML methods for general learning on bio-test data (T3) for all 17 datasets

| Dataset | Method | accuracy | recall | precision | F-measure | MCC | ROC-scores |
|---|---|---|---|---|---|---|---|
| BLCA | DEGnext | 98.42 | 98.42 | 98.49 | 0.98 | 0.97 | 0.99 |
| | DTC | 84.65 | 84.65 | 86.08 | 0.85 | 0.69 | 0.88 |
| | KNN | 90.18 | 90.18 | 90.21 | 0.9 | 0.79 | 0.94 |
| | RFC | 90.58 | 90.58 | 90.77 | 0.91 | 0.8 | 0.96 |
| | SVC | 85.08 | 85.08 | 86.25 | 0.85 | 0.69 | 0.94 |
| | XGBoost | 92.93 | 92.93 | 93.09 | 0.93 | 0.85 | 0.99 |
| BRCA | DEGnext | 98.8 | 98.8 | 98.83 | 0.99 | 0.98 | 0.99 |
| | DTC | 76.54 | 76.54 | 77.75 | 0.76 | 0.52 | 0.83 |
| | KNN | 89.77 | 89.77 | 89.84 | 0.9 | 0.79 | 0.96 |
| | RFC | 94.93 | 94.93 | 94.96 | 0.95 | 0.9 | 0.98 |
| | SVC | 82.81 | 82.81 | 86.68 | 0.82 | 0.67 | 0.99 |
| | XGBoost | 96.52 | 96.52 | 96.55 | 0.97 | 0.93 | 0.99 |
| CHOL | DEGnext | 100 | 100 | 100 | 1 | 1 | 0.99 |
| | DTC | 91.47 | 91.47 | 91.4 | 0.91 | 0.71 | 0.83 |
| | KNN | 99.28 | 99.28 | 99.29 | 0.99 | 0.98 | 0.99 |
| | RFC | 98.55 | 98.55 | 98.57 | 0.99 | 0.95 | 0.99 |
| | SVC | 90.76 | 90.76 | 91.77 | 0.9 | 0.69 | 0.99 |
| | XGBoost | 98.36 | 98.36 | 98.38 | 0.98 | 0.95 | 0.99 |
| COAD | DEGnext | 99.64 | 99.64 | 99.65 | 1 | 0.99 | 0.99 |
| | DTC | 75.12 | 75.12 | 76.58 | 0.75 | 0.51 | 0.81 |
| | KNN | 95.78 | 95.78 | 95.83 | 0.96 | 0.91 | 0.99 |
| | RFC | 96.35 | 96.35 | 96.39 | 0.96 | 0.93 | 0.99 |
| | SVC | 92.5 | 92.5 | 93.67 | 0.92 | 0.86 | 0.99 |
| | XGBoost | 97.93 | 97.93 | 97.94 | 0.98 | 0.96 | 0.99 |
| ESCA | DEGnext | 97.95 | 97.95 | 98.1 | 0.98 | 0.96 | 0.99 |
| | DTC | 77.08 | 77.08 | 80.64 | 0.76 | 0.5 | 0.79 |
| | KNN | 92.22 | 92.22 | 92.53 | 0.92 | 0.82 | 0.95 |
| | RFC | 91.2 | 91.2 | 91.54 | 0.91 | 0.8 | 0.96 |
| | SVC | 76.73 | 76.73 | 82.93 | 0.71 | 0.44 | 0.94 |
| | XGBoost | 93.76 | 93.76 | 94.31 | 0.94 | 0.86 | 0.97 |
| HNSC | DEGnext | 99.32 | 99.32 | 99.34 | 0.99 | 0.98 | 0.99 |
| | DTC | 78.88 | 78.88 | 79.09 | 0.79 | 0.51 | 0.77 |
| | KNN | 89.5 | 89.5 | 89.73 | 0.89 | 0.75 | 0.95 |
| | RFC | 94.28 | 94.28 | 94.44 | 0.94 | 0.87 | 0.98 |
| | SVC | 75.88 | 75.88 | 82.22 | 0.7 | 0.41 | 0.97 |
| | XGBoost | 95.23 | 95.23 | 95.34 | 0.95 | 0.89 | 0.99 |
| KICH | DEGnext | 100 | 100 | 100 | 1 | 1 | 0.99 |
| | DTC | 83.39 | 83.39 | 84.81 | 0.82 | 0.6 | 0.82 |
| | KNN | 96.69 | 96.69 | 96.76 | 0.97 | 0.92 | 0.97 |
| | RFC | 94.77 | 94.77 | 95.23 | 0.95 | 0.88 | 0.99 |
| | SVC | 90.17 | 90.17 | 91.15 | 0.9 | 0.77 | 0.99 |
| | XGBoost | 95.12 | 95.12 | 95.2 | 0.95 | 0.89 | 0.99 |
| KIRC | DEGnext | 99.78 | 99.78 | 99.78 | 1 | 1 | 0.99 |
| | DTC | 74.51 | 74.51 | 75.07 | 0.74 | 0.49 | 0.77 |
| | KNN | 87.47 | 87.47 | 88.19 | 0.87 | 0.75 | 0.95 |
| | RFC | 92.75 | 92.75 | 92.98 | 0.93 | 0.86 | 0.97 |
| | SVC | 71.65 | 71.65 | 79.41 | 0.68 | 0.48 | 0.95 |
| | XGBoost | 92.75 | 92.75 | 92.85 | 0.93 | 0.85 | 0.98 |
| KIRP | DEGnext | 100 | 100 | 100 | 1 | 1 | 0.99 |
| | DTC | 74.77 | 74.77 | 75.81 | 0.74 | 0.48 | 0.76 |
| | KNN | 88.15 | 88.15 | 89.91 | 0.88 | 0.77 | 0.96 |
| | RFC | 91.43 | 91.43 | 92.1 | 0.91 | 0.83 | 0.98 |
| | SVC | 80.34 | 80.34 | 85.33 | 0.79 | 0.63 | 0.95 |
| | XGBoost | 92.87 | 92.87 | 93.33 | 0.93 | 0.86 | 0.98 |
| LIHC | DEGnext | 95.93 | 95.93 | 96.23 | 0.96 | 0.85 | 0.96 |
| | DTC | 89.18 | 89.18 | 88.92 | 0.87 | 0.46 | 0.76 |
| | KNN | 93.99 | 93.99 | 94.03 | 0.93 | 0.73 | 0.93 |
| | RFC | 96.66 | 96.66 | 96.65 | 0.97 | 0.85 | 0.98 |
| | SVC | 88.85 | 88.85 | 90.19 | 0.85 | 0.41 | 0.97 |

*Continue on the next page*

Table S1: ROC scores of DEGnext and ML methods for general learning on bio-test data (T3) for all 17 datasets (cont.).

| Dataset | Method | accuracy | recall | precision | F-measure | MCC | ROC-scores |
|---|---|---|---|---|---|---|---|
| | XGBoost | 96.46 | 96.46 | 96.44 | 0.96 | 0.84 | 0.99 |
| LUAD | DEGnext | 99.82 | 99.82 | 99.83 | 1 | 1 | 0.99 |
| | DTC | 79.68 | 79.68 | 80.18 | 0.8 | 0.54 | 0.82 |
| | KNN | 89.26 | 89.26 | 89.4 | 0.89 | 0.75 | 0.94 |
| | RFC | 93.63 | 93.63 | 93.62 | 0.94 | 0.85 | 0.97 |
| | SVC | 82.54 | 82.54 | 86.32 | 0.8 | 0.61 | 0.98 |
| | XGBoost | 95.06 | 95.06 | 95.22 | 0.95 | 0.89 | 0.98 |
| LUSC | DEGnext | 99.88 | 99.88 | 99.88 | 1 | 1 | 0.99 |
| | DTC | 78.66 | 78.66 | 80.63 | 0.79 | 0.58 | 0.83 |
| | KNN | 94.16 | 94.16 | 94.47 | 0.94 | 0.88 | 0.98 |
| | RFC | 96.31 | 96.31 | 96.4 | 0.96 | 0.92 | 0.99 |
| | SVC | 90.47 | 90.47 | 92.2 | 0.9 | 0.81 | 0.99 |
| | XGBoost | 97.85 | 97.85 | 97.9 | 0.98 | 0.96 | 0.99 |
| PRAD | DEGnext | 99.35 | 99.35 | 99.36 | 0.99 | 0.99 | 0.99 |
| | DTC | 77.78 | 77.78 | 79.42 | 0.77 | 0.56 | 0.84 |
| | KNN | 93.7 | 93.7 | 93.79 | 0.94 | 0.87 | 0.98 |
| | RFC | 94.35 | 94.35 | 94.42 | 0.94 | 0.89 | 0.98 |
| | SVC | 84.81 | 84.81 | 86.87 | 0.85 | 0.72 | 0.96 |
| | XGBoost | 95.28 | 95.28 | 95.36 | 0.95 | 0.91 | 0.99 |
| READ | DEGnext | 95.39 | 95.39 | 96.54 | 0.95 | 0.92 | 0.99 |
| | DTC | 88.65 | 88.65 | 89.12 | 0.89 | 0.78 | 0.89 |
| | KNN | 88.8 | 88.8 | 89.35 | 0.89 | 0.78 | 0.95 |
| | RFC | 88 | 88 | 89.52 | 0.88 | 0.78 | 0.96 |
| | SVC | 79.54 | 79.54 | 83.54 | 0.79 | 0.63 | 0.93 |
| | XGBoost | 92 | 92 | 92.57 | 0.92 | 0.84 | 0.98 |
| STAD | DEGnext | 96.89 | 96.89 | 97.06 | 0.97 | 0.93 | 0.99 |
| | DTC | 78.35 | 78.35 | 78.23 | 0.77 | 0.48 | 0.76 |
| | KNN | 88.93 | 88.93 | 90.19 | 0.88 | 0.75 | 0.96 |
| | RFC | 89.69 | 89.69 | 89.72 | 0.9 | 0.76 | 0.95 |
| | SVC | 71.15 | 71.15 | 77.64 | 0.62 | 0.27 | 0.97 |
| | XGBoost | 93.81 | 93.81 | 94.06 | 0.94 | 0.86 | 0.98 |
| THCA | DEGnext | 99.87 | 99.87 | 99.87 | 1 | 1 | 0.99 |
| | DTC | 76.24 | 76.24 | 76.45 | 0.75 | 0.46 | 0.74 |
| | KNN | 92.41 | 92.41 | 92.8 | 0.92 | 0.83 | 0.96 |
| | RFC | 96.37 | 96.37 | 96.43 | 0.96 | 0.92 | 0.99 |
| | SVC | 86.54 | 86.54 | 88.5 | 0.86 | 0.71 | 0.98 |
| | XGBoost | 97.89 | 97.89 | 97.91 | 0.98 | 0.95 | 0.99 |
| UCEC | DEGnext | 99.6 | 99.6 | 99.61 | 1 | 0.99 | 0.99 |
| | DTC | 78.08 | 78.08 | 78.4 | 0.78 | 0.56 | 0.82 |
| | KNN | 90.59 | 90.59 | 91.15 | 0.91 | 0.82 | 0.97 |
| | RFC | 95.6 | 95.6 | 95.68 | 0.96 | 0.91 | 0.99 |
| | SVC | 85.59 | 85.59 | 87.58 | 0.86 | 0.73 | 0.97 |
| | XGBoost | 97.3 | 97.3 | 97.33 | 0.97 | 0.95 | 0.99 |

Table S2: ROC scores of DEGnext and ML methods for transfer learning on 100% of bio data (Q) for 8 testing datasets, namely BLCA, CHOL, COAD, ESCA, HNSC, KICH, READ, and STAD

| Dataset | Method | accuracy | recall | precision | F-measure | MCC | ROC-scores |
|---|---|---|---|---|---|---|---|
| BLCA | DEGnext | 95.69 | 95.69 | 95.76 | 0.96 | 0.91 | 0.95 |
| | DTC | 88.58 | 88.58 | 89.03 | 0.88 | 0.76 | 0.96 |
| | KNN | 91.34 | 91.34 | 91.35 | 0.91 | 0.82 | 0.98 |
| | RFC | 99.61 | 99.61 | 99.61 | 1 | 0.99 | 1 |
| | SVC | 90.94 | 90.94 | 91.08 | 0.91 | 0.81 | 0.98 |
| | XGBoost | 99.21 | 99.21 | 99.22 | 0.99 | 0.98 | 1 |
| CHOL | DEGnext | 98.26 | 98.26 | 98.49 | 0.98 | 0.94 | 0.99 |
| | DTC | 94.2 | 94.2 | 94.13 | 0.94 | 0.81 | 0.88 |
| | KNN | 99.82 | 99.82 | 99.82 | 1 | 0.99 | 1 |
| | RFC | 99.82 | 99.82 | 99.82 | 1 | 0.99 | 1 |
| | SVC | 91.3 | 91.3 | 92.16 | 0.9 | 0.71 | 1 |
| | XGBoost | 100 | 100 | 100 | 1 | 1 | 1 |
| COAD | DEGnext | 84.21 | 84.21 | 88.44 | 0.84 | 0.72 | 0.86 |
| | DTC | 78.98 | 78.98 | 80.57 | 0.79 | 0.59 | 0.84 |
| | KNN | 97.64 | 97.64 | 97.65 | 0.98 | 0.95 | 1 |
| | RFC | 99.36 | 99.36 | 99.36 | 0.99 | 0.99 | 1 |
| | SVC | 96.35 | 96.35 | 96.57 | 0.96 | 0.93 | 1 |
| | XGBoost | 99.79 | 99.79 | 99.79 | 1 | 1 | 1 |
| ESCA | DEGnext | 92.97 | 92.97 | 94.68 | 0.93 | 0.61 | 0.93 |
| | DTC | 91.71 | 91.71 | 92.62 | 0.91 | 0.82 | 0.93 |
| | KNN | 94.82 | 94.82 | 94.89 | 0.95 | 0.88 | 0.99 |
| | RFC | 99.48 | 99.48 | 99.49 | 0.99 | 0.99 | 1 |
| | SVC | 91.19 | 91.19 | 91.89 | 0.91 | 0.8 | 0.99 |
| | XGBoost | 99.48 | 99.48 | 99.49 | 0.99 | 0.99 | 1 |
| HNSC | DEGnext | 98.44 | 98.44 | 98.49 | 0.98 | 0.96 | 0.98 |
| | DTC | 82.43 | 82.43 | 83.5 | 0.83 | 0.61 | 0.84 |
| | KNN | 92.37 | 92.37 | 92.33 | 0.92 | 0.82 | 0.98 |
| | RFC | 99.46 | 99.46 | 99.46 | 0.99 | 0.99 | 1 |
| | SVC | 76.57 | 76.57 | 82.53 | 0.71 | 0.43 | 0.99 |
| | XGBoost | 99.46 | 99.46 | 99.46 | 0.99 | 0.99 | 1 |
| KICH | DEGnext | 98.75 | 98.75 | 98.79 | 0.99 | 0.97 | 0.98 |
| | DTC | 88.24 | 88.24 | 88.33 | 0.88 | 0.72 | 0.87 |
| | KNN | 98.37 | 98.37 | 98.37 | 0.98 | 0.96 | 1 |
| | RFC | 99.67 | 99.67 | 99.68 | 1 | 0.99 | 1 |
| | SVC | 92.81 | 92.81 | 93.49 | 0.93 | 0.83 | 0.99 |
| | XGBoost | 99.67 | 99.67 | 99.68 | 1 | 0.99 | 1 |
| READ | DEGnext | 86.05 | 86.05 | 89.23 | 0.86 | 0.75 | 0.87 |
| | DTC | 95.87 | 95.87 | 95.97 | 0.96 | 0.92 | 0.96 |
| | KNN | 95.04 | 95.04 | 95.04 | 0.95 | 0.9 | 0.99 |
| | RFC | 99.17 | 99.17 | 99.19 | 0.99 | 0.98 | 1 |
| | SVC | 76.03 | 76.03 | 80.71 | 0.75 | 0.57 | 0.95 |
| | XGBoost | 100 | 100 | 100 | 1 | 1 | 1 |
| STAD | DEGnext | 97.77 | 97.77 | 97.89 | 0.98 | 0.95 | 0.96 |
| | DTC | 85.31 | 85.31 | 85.21 | 0.85 | 0.65 | 0.83 |
| | KNN | 92.78 | 92.78 | 93.34 | 0.93 | 0.84 | 0.99 |
| | RFC | 98.45 | 98.45 | 98.45 | 0.98 | 0.96 | 1 |
| | SVC | 82.22 | 82.22 | 85.92 | 0.8 | 0.6 | 0.99 |
| | XGBoost | 99.74 | 99.74 | 99.74 | 1 | 0.99 | 1 |
