# Supplementary material for: DEGnext: classification of differentially expressed genes from RNA-seq data using a convolutional neural network with transfer learning
Source: BMC Bioinformatics. 2022 Jan 6;23:17. doi: 10.1186/s12859-021-04527-4 (PMC8734099; doi:10.1186/s12859-021-04527-4)
Supplement: Supplementary file 2 — Additional file 2. Readme. [file 12859_2021_4527_MOESM2_ESM.pdf]

DEGnext

Tulika Kakati

July 06, 2021

Citation

Please use the source code ethically.

Prerequisites for testing platform

Python 3.6.1 or above

pytorch (1.3 and above), sklearn (0.21.3 and above)

#Installation

Supported Operating Systems: Windows 10, Mac OS (10.10.x+), Unix (Ubuntu, Other).

a.) Download and install Anaconda (choose the latest Python version) from https://docs.anaconda.com/anaconda/install/windows/. DEGnext was implemented and tested with Python version 3.6.1 on Windows 10.

b.) To install pytorch, type the following commands in Anaconda prompt

```
conda install pytorch -c pytorch

pip install torchvision

pip install sklearn

pip install matplotlib

pip install pickle

pip install xgboost

pip install pandas
```

c.) IDE: Jupyter Notebook or PyCharm or Spyder (https://docs.anaconda.com/anaconda/user-guide/getting-started/; https://docs.anaconda.com/anaconda/user-guide/getting-started/;)

Introduction

DEGnext is a CNN model, to predict upregulating (UR) and downregulating (DR) genes from gene expression data of 17 cancer datasets obtained from The Cancer Genome Atlas (TCGA) database. DEGnext uses biologically validated data along with logarithmic fold change (logFC) values to classify

differentially expressed genes (DEGs) as UR and DR genes. We applied transfer learning to our model to leverage the knowledge of trained feature maps to untrained cancer datasets.

Datasets

Here, we show the effectiveness of our model on 17 best fold cancer datasets. Each dataset is split as non-bio train data (T1), non-bio test data (T2), fine-tune data (F1), and bio-test data (T3).

In the paper, we show that DEGnext performs competitively well other machine learning (ML) methods even with 5-fold cross validation.

General Learning:

Here, we have two experiments.

In the first experiment, we used all the 17 cancer datasets to train on non-bio train data (T1). Since the T1 data has three labels “0”, “1”, and “2”, this training is for a three-class problem. DEGnext uses CrossEntropyLoss() as the loss function and optim.Adam() as the optimizer to compute the cross entropy loss between input and output and updates the parameters based on the backpropagated loss gradients. For predicted classes “0”, “1”, and “2”, the input gene is classified as DR, UR and neutral gene, respectively.

For the second experiment, we first fine-tune the model from experiment 1 on F1 data. Since F1 data has 0 and 1 labels, the second level of training is a two-class problem. Here, we used the BCEWithLogitsLoss() loss function to fine tune the model. After fine-tuning, DEGnext model is then tested using bio-test data (T3). The second level of training incorporates both prior disease-related biological knowledge and log2FC estimates (sample variance) of the data to the CNN model, which enables capture of non-linear gene expression patterns and enhances prediction performance of the model in determining UR and DR genes. The major advantage of our CNN model is that it allows efficient transfer learning by reusing the feature-map signatures learned from the trained model.

Transfer learning:

For transfer learning, we used 9 datasets as training datasets: "TCGA-BRCA","TCGA-KIRC","TCGA-KIRP","TCGA-LIHC","TCGA-LUAD","TCGA-LUSC","TCGA-PRAD","TCGA-THCA","TCGA-UCEC". We used 8 datasets as testing or untrained datasets: ["TCGA-BLCA","TCGA-CHOL","TCGA-COAD","TCGA-ESCA","TCGA-HNSC","TCGA-KICH","TCGA-READ","TCGA-STAD"]. First, we trained on non-bio train data (T1) for all 9 training datasets sequentially. For training on T1, since there are three labels, “0”, “1”, and “2”, we use CrossEntropyLoss() as the loss function and optim.Adam() as the optimizer, with a batch size of 256. For fine tuning the model, we modify the output layer L5 and remove the final softmax layer to classify the DEGs as “0”, or “1”. We used the BCEWithLogitsLoss() loss

function to fine tune the model again with the F1 data for all 9 training datasets. We used the pretrained model as a feature extractor and leveraged the knowledge acquired from the trained model to predict UR and DR genes from bio-test data of 8 untrained datasets.

Pre-requisites to run DEGnext

1. Please create a folder "DEGnext " in your preferred directory. Please download the "datasets" folder from shared link https://drive.google.com/drive/u/0/folders/1Q43A8DKR-iumhgp5LxtQg1wWUcX80Vro . Unzip the folder and copy the "datasets" folder to "DEGnext " folder.

2. Inside "DEGnext " folder, create two sub-folders: one as "general-learning" and the other as "transfer-learning".

3. For general learning, download and unzip the source files from Additional File 3 to "general-learning" folder. From a terminal window, go to the general-learning directory. To run the first experiment, type “python DEGnext _exp_1.py”. To run the second experiment, type “python DEGnext _exp_2.py”.

4. For transfer learning, download and unzip the source files from Additional File 4 to "transfer-learning" folder. From a terminal window, go to the transfer-learning directory. To run the transfer learning, type “python DEGnext .py”.

5. If you want to test the model on a two-class classification problem without training and fine-tuning, please download and unzip the source files from Additional File 5. From a terminal window, go to the test-case directory. To run DEGnext for an input file, type “python DEGnext .py”.

Output of DEGnext

Here, I show the output of **DEGnext .py** using an example input file.

The test result for the input dataset can be found in a file **“Bio_test_data_accuracy_selected_datasets.txt”** in terms of accuracy, recall, precision, fmeasure, and mcc scores.

The predicted upregulating and downregulating genes can be found in a file **Predicted_UR_and_DR_genes.txt**

User may perform the functional analysis, such as pathway analysis of these UR and DR genes to find their association to progression of cancer.
